# Vitamin D (1,25(OH)_2_D3) induces α-1-antitrypsin synthesis by CD4^+^ T cells, which is required for 1,25(OH)_2_D3-driven IL-10

**DOI:** 10.1016/j.jsbmb.2019.01.014

**Published:** 2019-05

**Authors:** Sarah Dimeloe, Louise V. Rice, Hebe Chen, Charlotte Cheadle, John Raynes, Paul Pfeffer, Paul Lavender, David F. Richards, Mun Peak Nyon, James M. McDonnell, Claudia Kemper, Bibek Gooptu, Catherine M. Hawrylowicz

**Affiliations:** a MRC and Asthma UK Centre for Allergic Mechanisms of Asthma, King’s College London, Guy’s Hospital, London, SE1 9RT, United Kingdom; b Immunology and Infection Department, London School of Hygiene and Tropical Medicine, London, WC1E 7HT, United Kingdom; c Institute of Structural and Molecular Biology/Crystallography, Department of Biological Sciences, Birkbeck College, University of London, Malet Street, London, WC1E 7HX, United Kingdom; d MRC Centre for Transplantation, Division of Transplantation Immunology and Mucosal Biology, King’s College London, Guy’s Hospital, London, SE1 9RT, United Kingdom; e NIHR Leicester BRC-Respiratory and Leicester Institute of Structural & Chemical Biology, Glenfield Hospital, Groby Road, Leicester, LE3 9QP, United Kingdom

**Keywords:** Immune regulation, IL-10, α-1-Antitrypsin, Complement, C3a

## Abstract

Studies to identify novel immune-regulatory functions of active vitamin D (1,25(OH)_2_D3) in human CD4^+^ T cells revealed that 1,25(OH)_2_D3 potently induced expression of the gene *SERPINA1*, encoding the anti-protease α-1-antitrypsin. We confirmed α-1-antitrypsin protein expression by 1,25(OH)_2_D3-treated CD4^+^ T cells, but not in CD8^+^ T cells or monocytes. α-1-Antitrypsin promotes anti-inflammatory IL-10 synthesis in other immune cell populations. We therefore investigated its immune-regulatory effects in CD4^+^ T cells. Plasma-derived α-1-antitrypsin drove IL-10 synthesis by CD4^+^ T cells, which was not dependent on anti-protease activity, but appeared to require a serum-binding factor, since this could not be achieved with recombinant protein. α-1-Antitrypsin is reported to bind complement components, which regulate T cell function. A role for this interaction was therefore probed. Plasma-derived, but not recombinant α-1-antitrypsin contained C3a. Surface Plasmon Resonance and Microscale Thermophoresis demonstrated α-1-antitrypsin binding to C3a. Addition of C3a to CD4^+^ T cells cultured with recombinant α-1-antitrypsin restored induction of IL-10, whereas neutralisation of C3a abrogated IL-10 induced by plasma-derived α-1-antitrypsin. To interrogate an endogenous role for the α-1-antitrypsin-C3a axis in 1,25(OH)_2_D3-driven CD4^+^ T cell IL-10 synthesis, we treated cells from healthy or α-1-antitrypsin-deficient individuals (which transcribe *SERPINA1* but do not secrete protein) with 1,25(OH)_2_D3. A significant correlation was identified between *SERPINA1* and *IL10* gene expression in healthy donor CD4^+^ T cells, which was absent in cells from α-1-antitrypsin-deficient individuals. Therefore, α-1-antitrypsin is required for 1,25(OH)_2_D3-induced IL-10 expression in CD4^+^ T cells, interacting with C3a to drive IL-10 expression.

## Highlights

• Human CD4^+^ T cells exposed to 1,25(OH)_2_D3 secrete α-1-antitrypsin – representing a *novel cellular source* of this protein.
• α-1-Antitrypsin promotes IL-10 secretion by human CD4^+^ T cells, via direct interaction with complement C3a.
• 1,25(OH)_2_D3 is unable to increase IL10 transcription in CD4^+^ T cells from α-1-antitrypsin-deficient individuals.
• Therefore, autocrine α-1-Antitrypsin is required for 1,25(OH)_2_D3-driven IL-10 expression.

## 1 Introduction

Vitamin D is a potent anti-inflammatory mediator, with its active form (1,25-dihydroxyvitamin D, 1,25(OH)_2_D3) having a range of immune-regulatory functions including direct induction of anti-microbial functions and the generation of tolerogenic antigen presenting cells and regulatory T cells [1]. Effects of 1,25(OH)_2_D3 on CD4^+^ ‘helper’ T cells, which co-ordinate the immune response have been a particular interest of our laboratory and previous work has identified 1,25(OH)_2_D3-mediated induction of the anti-inflammatory cytokine IL-10, the immune-suppressive ligand CD200 and the regulatory T cell hallmark transcription factor FoxP3 [[2], [3], [4], [5]]. The induction of inhibitory antigens such as CTLA4, first described by Jeffery et al. [6], occur rapidly in culture and likely represent direct transcriptional regulation by 1,25(OH)_2_D3. In contrast, evidence exists that 1,25(OH)_2_D3 controls synthesis of the anti-inflammatory cytokine IL-10, with positive correlations seen in vivo [4,7]. However the capacity of 1,25(OH)_2_D3 to induce IL-10 synthesis by CD4^+^ T cells occurs comparatively slowly in culture [4], and it seems probable that this may represent an indirect effect, requiring signals from additional cells and/or 1,25(OH)_2_D3-induced mediators.

Alpha-1-antitrypsin (α-1-antitrypsin) is a key inhibitor of neutrophil elastase with particular relevance in the airways. Variant alleles of α-1-antitrypsin predispose to conformational change in the protein, polymerisation and retention within the endoplasmic reticulum, and a concomitant deficiency of protein. Its key anti-protease role in the airway is evidenced by the fact that α-1-antitrypsin-deficiency represents the only established heritable cause of chronic obstructive pulmonary disease (COPD) caused by excessive elastolytic activity and tissue damage [8]. However, native α-1-antitrypsin additionally has independent anti-inflammatory properties, with reported effects in vitro on neutrophil chemotaxis, monocyte and dendritic cell maturation and cytokine profiles [9]. Consistent with this, in animal models of autoimmunity and transplantation, human α-1-antitrypsin is protective, with evidence for induction of immune-regulatory pathways [10,11]. Associations exist between reduced α-1-antitrypsin expression and/or function and a number of human diseases of immune dysregulation [10]. As yet these properties of α-1-antitrypsin are not fully understood, but the cytokine IL-10 may play a key role. Human innate immune cells produce IL-10 in response to α-1-antitrypsin in vitro, COPD progression in α-1-antitrypsin deficiency associates with IL-10 polymorphisms and α-1-antitrypsin replacement therapy increases circulating IL-10 in those individuals [12].

This study investigated whether vitamin D (1,25(OH)_2_D3) controls gene and protein expression for α-1-antitrypsin in human peripheral T cells. Subsequent studies investigated whether this represents a direct action of α-1-antitrypsin, or whether additional co-factors are required, as well as whether α-1-antitrypsin may be required for the immune regulatory functions, specifically IL-10 induction, of 1,25(OH)_2_D3.

## 2 Materials and methods

### 2.1 Patient details

Peripheral blood was obtained from healthy volunteers or individuals with diagnosed α-1-antitrypsin deficiency (PiZZ) attending the London Alpha-1 Antitrypsin Deficiency Service, UCL/The Royal Free Hospital. All volunteers signed a consent form and all studies were fully approved by the NRES research committee (London) (REC reference 14/LO/1699 for healthy donors and 13/LO/1085 for α-1-antitrypsin-deficient individuals).

For the data in Fig. 5 volunteer demographics were: Healthy subjects (aged 25–62, 5:4 female:male ratio); α-1-antitrypsin-deficient (PiZZ) individuals (25–74 years, 3:7 female:male ratio). PiZZ individuals had not received α-1-antitrypsin replacement therapy.

### 2.2 Cell purification and culture

Cell purification from peripheral blood was performed as previously described [4]. Purified T cells (1 × 10^6^ cells/ml, in AIM-V serum-free medium, or RMPI medium plus 10% foetal calf serum (FCS); both Life Technologies Ltd.– indicated in the figure legend) were stimulated with 1 μg/ml plate-bound anti-CD3 (OKT-3, in-house) and 2 μg/ml plate-bound anti-CD28 (CD28.2, BD Biosciences, San Diego, CA) and 50U/ml IL-2 (Eurocetus, Harefield, UK). They were treated with α-1-antitrypsin (at concentrations indicated), C3a (100 ng/ml, Complement Technology, Inc., Tyler, TX) or a titration of 1,25(OH)_2_D3 (10^−9^–10^−7^ M, Enzo Life Sciences, USA). α-1-Antitrypsin protein samples were obtained commercially (Sigma-Aldrich, UK) or purified in-house from human plasma or an *E. coli* recombinant system. Other sources of glycosylated recombinant protein were not used, since the functional experiments with *E. coli* generated protein identified glycosylation not to be required. In-house preparations were as described previously [13,14] but with a modification to the plasma protein purification in which the initial chromatography step was performed using an alpha-select (GE Heathcare, US) column rather than thiol exchange. Additionally in some cultures, neutralising anti-C3a (5 μg/ml, clone 2991, gift from Professor Joerg Koehl, Lubeck University, Germany) was included. Supernatants were harvested at time points indicated for analysis of cytokines and α-1-antitrypsin. Cell pellets were harvested at 14 for qPCR analysis of SERPINA1 gene expression. Purified monocytes (1 × 10^6^ cells/ml, in AIM-V serum-free medium, or RMPI medium plus 10% foetal calf serum; both Life Technologies Ltd) were unstimulated or stimulated with lipopolysaccharide (LPS, 500 ng/ml, Sigma-Aldrich, UK) for 48 h in the absence or presence of 1,25(OH)_2_D3 as indicated. Supernatants were harvested at 48 h for α-1-antitrypsin analysis.

### 2.3 Cytokine analysis

Cytokine concentrations in cell culture supernatants were assessed using the cytometric bead array (CBA) flex-set, according to the manufacturer’s instructions and using AIM-V serum-free medium as a diluent for standards (1 in 5 dilution of reagents, validated). Samples were assayed using the BDFortessa or FACSCalibur flow cytometer (BD, UK). Data were analysed using FlowJo (version 9.2, TreeStar Inc) and GraphPad Prism (version 5 for Mac OS X, GraphPad Software Inc.). The lower limit of detection for all cytokines was 1.5 pg/ml.

### 2.4 qPCR

qPCR (real time RT-PCR) was performed as previously described [4], in triplicate, using an Applied Biosystems 7900 HT system and a FAM-labelled Assay-on-Demand reagent set for SERPINA1 : Hs01097800_m1. Real time RT-PCR reactions were multiplexed using VIC labelled 18 s primers and probes (Hs99999901_s1) as an endogenous control and analyzed using SDS software version 2.1 (Applied Biosystems), according to the 2-(ΔΔCt) method.

### 2.5 Alpha-1-antitrypsin ELISA

The α-1-antitrypsin ELISA employed a commercial polyclonal rabbit anti-human α-1-antitrypsin primary antibody (DAKO, Cambridgeshire, UK) and a biotinylated affinity-purified rabbit anti-human α-1-antitrypsin secondary antibody. This was purified from rabbit antiserum (Dade Behring, Marburg, Germany) using antitrypsin coupled to activated CH-Sepharose 4B beads (Amersham Bioscience, Pittsburgh, USA) and conjugated with biotin using Pierce EZ-link NHS-LC Biotin^®^ (Pierce, Rockford, Illinois, USA). α-1-Antitrypsin standards were prepared in RPMI medium plus 10% foetal calf serum, by serial 1:2 dilutions from the top standard (200 ng/ml; prepared from human plasma-purified α-1-antitrypsin, Sigma-Aldrich). ExtrAvidin^®^ alkaline phosphatase enzyme (Sigma-Aldrich) and phospho-nitrophenylphosphate substrate (Sigma-Aldrich) were used as a detection system. α-1-Antitrypsin levels were measured using an Anthos HTII Plate reader (Anthos, UK) at an absorbance at 405 nm and quantified using GraphPad Prism software (version 5 for Mac OS X, GraphPad Software Inc.). The lower limit of detection for this assay was 0.32 ng/ml.

### 2.6 Chymotrypsin activity assay

1–5 nM α-1 antitrypsin was pre-incubated with 1 μM α-chymotrypsin from bovine pancreas (Sigma, UK) made up to a total volume of 100 μl with reaction buffer (0.03MNa2HPO4, 160 mM NaCl, 0.1% PEG4K, pH7.4). The substrate for chymotrypsin, 0.1 mM N-succinyl-Ala-Ala-Pro-Phe P-nitroanilide (Sigma, UK) was added and absorbance (405 nm) was read on the CARY UV spectrophotometer (Agilant Technologies, USA) at 0 min (T0) and after 10 min (T10) incubation at room temperature. Readings are plotted as normalised T10-T0 405 nm absorbance against α-1-antitrypsin concentration.

### 2.7 Surface plasmon resonance

SPR assays were conducted with a Biacore T200 instrument (GE Healthcare). Specific binding surfaces were prepared by coupling protein diluted in sodium acetate buffer, pH 4.5, to the dextran matrix of CM5 sensor chips using a Biacore Amine Coupling Kit. Biotinylated-C3a (Complement Technology Inc., Texas, US) was produced using a protocol designed to haved no more than one biotin molecule per molecule of C3a, by reacting protein with biotinamidohexanoic acid 3-sulfo*-N-*hydroxysuccinimide ester sodium salt (Sigma-Aldrich) at a 3:1 M ratio. When commercially produced α-1-antitrypsin was used it was dissolved to 10 mg/ml in phosphate buffered saline (PBS) and filtered using a 0.45 μm filter; size-exclusion chromatography (performed by Marie Pang) was used to remove any α-1-antitrypsin aggregates. Assays reported here typically used immobilization levels of 800–1500 resonance units. Samples of serially diluted α-1-antitrypsin were injected, and all measurements were performed at a continuous flow rate of 10 μl/minute in sterile Dulbecco’s PBS buffer. Regeneration of ligand-bound surfaces after binding of each potential ligand sample was achieved using a single 60 s injection of 2.0 M KCl. Non-specific binding was assessed by performing sample injections over a sensor surface that underwent identical activation and blocking but with no ligand immobilised. Nonspecific binding was subsequently subtracted from the specific reaction prior to analysis. Data were analysed using the Biaevaluation 3.1 analysis package (GE Healthcare).

### 2.8 Microscale thermophoresis

MST was performed using the Monolith NT.115 instrument (NanoTemper-Technologies, Munich, Germany), with LED settings at 70% and MST power at 20%. NT-647 labeled AAT (NanoTemper-Technologies, Munich, Germany) at 0.5 μM was incubated for 30 min with C3a at concentrations between 6 nM and 205 μM before loading in standard MST capillary tubes for binding measurements.

### 2.9 Statistics

Results are presented either as individual results of independent experiments or summarised as mean ± standard error of the mean deviation (SEM) for normally distributed data or median ± interquartile range (IQR) for non-normal data as indicated in the figure legend. Summarised paired data were statistically tested by paired or unpaired *t*-test or repeated measures ANOVA for normally distributed data, or using the Wilcoxon, Mann–Whitney *U* or Friedman test for non-normal data as indicated in specific figure legends. All p values were corrected for multiple comparisons using the Bonferroni test or Dunns test respectively. Differences were considered significant at the 95% confidence level. All statistical analyses were carried out using Graphpad Prism version 5.0 for Macintosh OS X.

## 3 Results

### 3.1 1,25(OH)_2_D3 induces α-1-antitrypsin expression by human CD4^+^ T cells

**Fig. 1:**
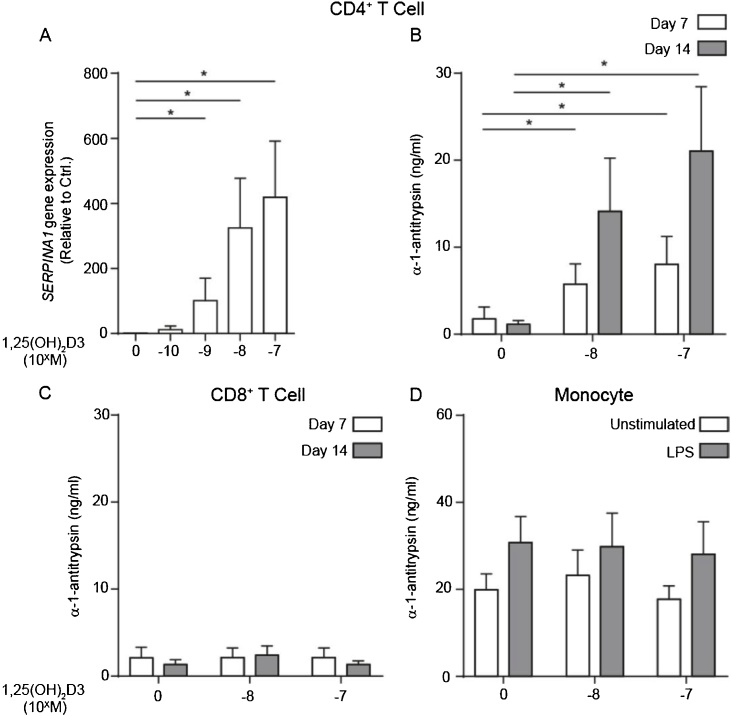
**1,25(OH)_2_D3 drives α-1-antitrypsin expression by human peripheral CD4^+^ T cells, but not CD8+ T cells or monocytes**. (a&b) CD4^+^ T cells, or (c) CD8^+^ T cells (1 × 10^6^ in 1 ml), in RPMI 10%FCS, were stimulated with anti-CD3 (1 μg/ml) and IL-2 (50 u/ml) for two rounds of seven days, in presence of 1,25(OH)_2_D3 as indicated (a: n = 9, b: n = 4, c: n = 4). (d) Human purified peripheral CD14^+^ monocytes (1 × 10^6^ in 1 ml), in RPMI 10%FCS, were stimulated with LPS (500 ng/ml) for 48 h in presence of 10^−7^ M 1,25(OH)_2_D3 as indicated (n = 4). α-1-Antitrypsin expression was assessed by (a) qPCR for SERPINA1 mRNA and (b–d) ELISA. mRNA quantification by qPCR was normalised to 18S transcription and is shown as a relative quantity (RQ) compared to expression under control conditions in vitro. Data are summarised as mean ± SEM. (a) p value derives from Friedman’s non-parametric repeated measures test. * p < 0.050 as assessed by Dunn’s paired post-test for multiple comparisons. (b) p values derive from a repeated measures ANOVA.

Preliminary studies to identify novel, unidentified immune-regulatory functions of 1,25(OH)_2_D3 in human CD4^+^ T cells, and to identify potential mediators of 1,25(OH)_2_D3-induced anti-inflammatory IL-10 expression, identified *SERPINA1*, encoding α-1-antitrypsin, as potently upregulated. qPCR analysis confirmed 1,25(OH)_2_D3-upregulation of *SERPINA1* (Fig. 1a) and ELISA confirmed α-1-antitrypsin protein secretion by these cells (Fig. 1b), although not by 1,25(OH)_2_D3-treated CD8^+^ T cells studied under the same experimental conditions (Fig. 1c). CD14^+^ monocytes produced α-1-antitrypsin constitutively and increased their secretion to similar levels to CD4^+^ T cells when stimulated with LPS –in agreement with previous reports [15]. However, monocytes did not respond to 1,25(OH)_2_D3 for further upregulation. (Fig. 1d). We have therefore identified that 1,25(OH)_2_D3 promotes α-1-antitrypsin synthesis by human CD4^+^ T cells – *a novel cellular source* of this protein.

### 3.2 Human plasma-derived α-1-antitrypsin increases CD4+ T cell IL-10 production

**Fig. 2:**
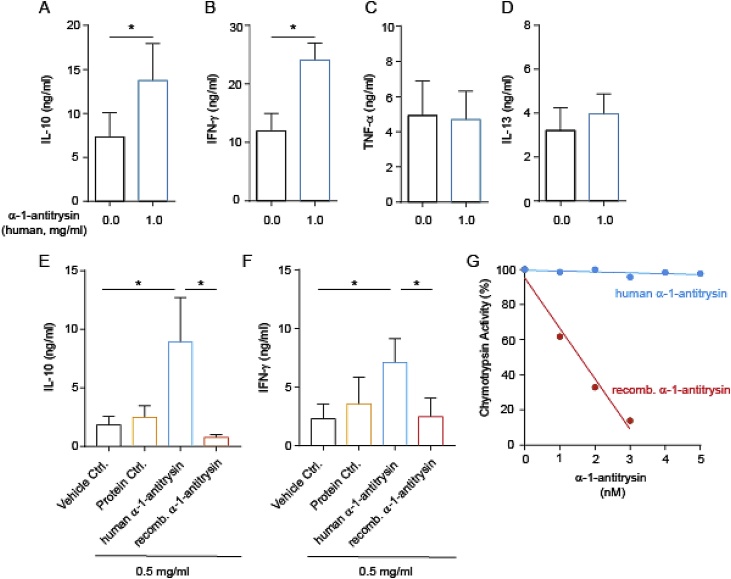
**Human plasma-derived, but not recombinant α-1-antitrypsin promotes production of IL-10 and IFN-γ by human CD4^+^ T cells, which is independent of anti-protease activity**. (a–f) CD4^+^ T cells (1 × 10^6^ in 1 ml) were stimulated with anti-CD3 (1 μg/ml) and IL-2 (50 u/ml) for 48 h without or with the indicated α-1-antitrypsin preparation or protein control. (a–d) Human α-1-antitrypsin was commercially obtained (e–f) human α-1-antitrypsin was lab-purified (see Methods for further details). Cytokine levels were assessed by CBA and are summarised as mean ± SEM. p values derive from (a–d, n = 8) paired *t*-tests and (e–f, n = 4) one-way ANOVA. * p < 0.05 and *** p < 0.001 as assessed by paired *t*-test (a–d) or Bonferroni’s paired post-test for multiple comparisons (e–f). (g) 0–5 nM α-1 antitrypsin was pre-incubated with chymotrypsin before the addition of 0.1 mM substrate *N*-succinyl-Ala-Ala-Pro-Phe P-nitroanilide and the inhibitory capacity of each α-1-antitrypsin preparation assed by the 405 nm absorbance. Data is presented as 405 nM absorbance T10-T0 normalised to 1 against α-1-antitrypsin concentration.

α-1-Antitrypsin is reported to have potent immune-regulatory effects in vivo, as well as on total peripheral blood mononuclear cells (PBMC) and innate immune cell populations [9]. We were therefore interested to define its effects on human CD4^+^ T cells, which we have identified produce this protein when stimulated in the presence of 1,25(OH)_2_D3 (Fig. 1). Initially, we assessed cytokine profiles of CD4^+^ T cells stimulated in the presence of α-1-antitrypsin purified from human plasma, using concentrations of α-1-antitrypsin similar to circulating levels (in vitro doses of 10–1000 μg/ml are equivalent to 0.18–18 μM; circulating levels are 20–48 μM). These experiments revealed that human plasma-derived α-1-antitrypsin significantly increased CD4^+^ T cell IL-10 secretion (Fig. 2a). Enhanced secretion of IFN-γ (Fig. 2b), but not TNF-α (Fig. 2c) or IL-13 (Fig. 2d) were also observed. CD4^+^ T cells responded to α-1-antitrypsin for induction of IL-10 in a dose-dependent manner from 10 to 1000 μg/ml with significant effects observed at 500 μg/ml and maximal effects observed at 1 mg/ml α-1-antitrypsin (Supplementary Fig. 1). Commercial preparations of human plasma-derived α-1-antitrypsin are known to contain non-native conformations of the protein that are inactive as protease inhibitors [16]. We therefore repeated our experiments, comparing human plasma α-1-antitrypsin with a recombinant α-1-antitrypsin preparation, as well as a control for exogenous protein addition (at the same concentration). These experiments interestingly revealed that human plasma-derived, *but not* recombinant α-1-antitrypsin induced IL-10 (Fig. 2E) and IFN-γ (Fig. 2F) secretion, whereas the protein control had little effect on cytokine levels. A chymotrypsin activity assay confirmed that human plasma-derived α-1-antitrypsin had very little anti-protease activity compared to the recombinant preparation (Fig. 2G). Taken together, these data suggest that the effects of α-1-antitrypsin on cytokine secretion do not require serine protease inhibitory activity, but may require a plasma-derived molecule binding to α-1-antitrypsin –not present in the recombinant preparation. We next explored this possibility.

### 3.3 α-1-Antitrypsin directly binds to complement C3a

**Fig. 3:**
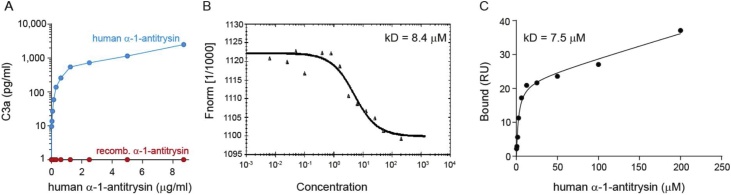
**α-1-Antitrypsin and C3a directly interact**. (a) C3a content in commercial human plasma-derived and recombinant α-1-antitrypsin was assessed by CBA anaphylatoxin flex set. (b–c) Direct physical interaction of α-1-antitrypsin with C3a was identified using (b) surface plasmon resonance (SPR) and (ii) microscale thermophoresis (MST) methodology. Curve fit analyses indicated Kd values of 8.4 μM (b) and 7.5 μM (c).

The complement system, and particularly the anaphylatoxins C3a and C5a are produced by immune cells and regulate cytokine secretion, particularly by CD4^+^ T cells [17]. Furthermore, α-1-antitrypsin has previously been reported to interact with total C3 [18]. We therefore explored whether these molecules may play a role in the induction of cytokine secretion by human plasma-derived α-1-antitrypsin. In initial experiments, we assessed whether C3a, C4a or C5a were present in the different α-1-antitrypsin preparations we employed. Indeed, C3a was detected in the human plasma-derived α-1-antitrypsin, but not the recombinant preparation (Fig. 3a). C4a and C5a were detectable at lower levels in the plasma-derived preparation only (Supplementary Fig. 2). The capacity of α-1-antitrypsin to directly interact with C3a was next assessed using biophysical methods. Specifically, the interaction between α-1-antitrypsin and C3a was assessed using (i) surface plasmon resonance (SPR), where randomly biotinylated C3a was immobilized via direct amine coupling to sensor chips and binding of α-1-antitrypsin detected by Biacore T200; and (ii) Microscale Thermophoresis (MST) performed using 0.5 μM NT-647 labelled recombinant α-1-antitrypsin and 205 μM–6 nM C3a. SPR studies demonstrated an interaction of α-1-antitrypsin with C3a (Fig. 3b), which was further validated by MST (Fig. 3c) with good agreement between K_d_ values determined by the two methods (7.53 μM and 8.4 μM respectively). Although this is a relatively moderate affinity binding interaction, it is plausible that it is physiologically relevant given the high circulating levels of α-1-antitrypsin of around 20–48 μM [19].

### 3.4 α-1-Antitrypsin and C3a interact to promote CD4^+^ T cell IL-10 secretion

**Fig. 4:**
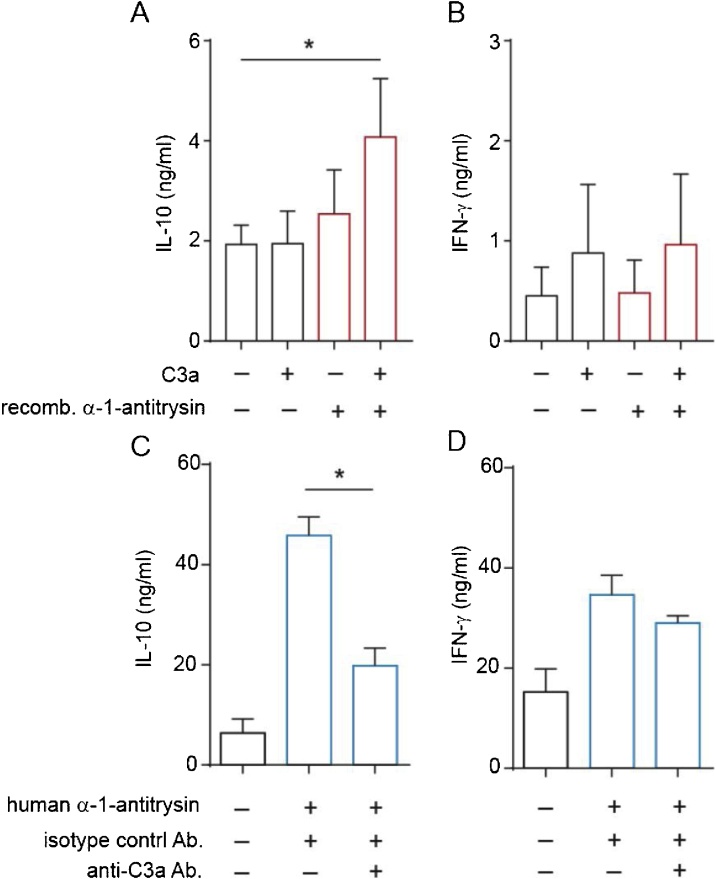
**C3a and α-1-antitrypsin cooperatively induce IL-10 in human CD4+ T cells**. CD4^+^ T cells were cultured for 48 h in serum-free medium, in the absence or presence of (a–b) recombinant α-1-antitrypsin (100 μg/ml) and/or C3a (100 ng/ml) as indicated or (c–d) commercial human plasma-derived α-1-antitrypsin (500 μg/ml) with or without a C3a neutralising or isotype control (anti-C3a/IgG1, 5 μg/ml) antibody. Cytokine secretion was assessed by CBA and presented as mean ± SEM. p values derived from a one-way ANOVA. * p < 0.05, ** p < 0.01 as assessed by Bonferroni’s paired post-test for multiple comparisons (n = 4).

To establish the role for C3a in α-1-antitrypsin-induced IL-10 in human CD4^+^ T cells, we undertook two complementary experimental approaches. Firstly, we added exogenous C3a to cultures containing recombinant α-1-antitrypsin. This resulted in significant IL-10 production (Fig. 4a), whilst not increasing IFN-γ levels (Fig. 4b). Secondly, C3a was neutralized with a specific antibody in cultures of CD4^+^ T cells stimulated in the presence of plasma- derived α-1-antitrypsin. These experiments demonstrated that inclusion of an anti-C3a specific antibody, but not an isotype control antibody significantly reduced IL-10 levels in (Fig. 4c), whilst again not affecting IFN-γ secretion. These experiments indicate that α-1-antitrypsin and C3a act in concert to drive IL-10 secretion by CD4^+^ T cells.

### 3.5 α-1-Antitrypsin is required for 1,25(OH)_2_D3-induced IL-10

**Fig. 5:**
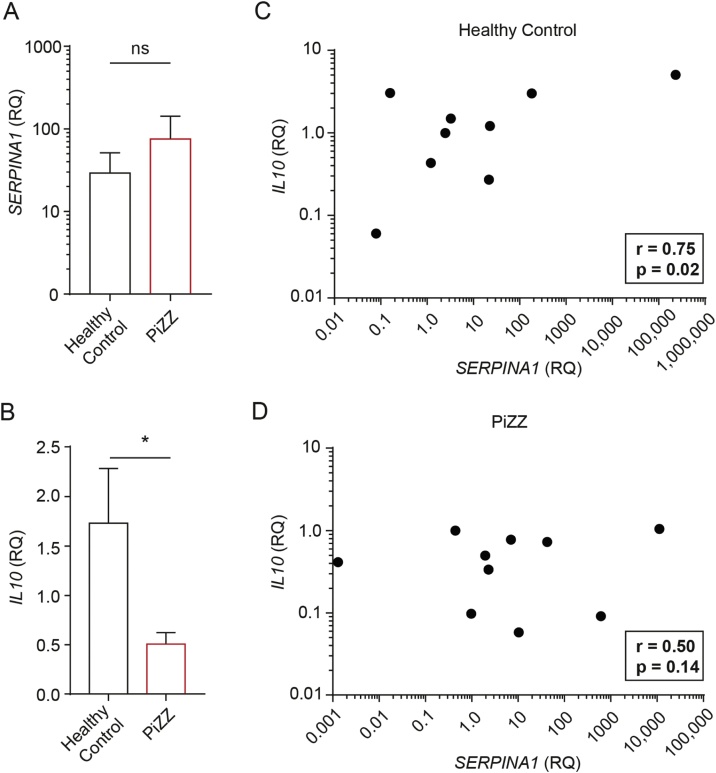
**α-1-Antitrypsin is required for 1,25(OH)_2_D3-induced IL-10 expression by CD4^+^ T cells**. CD4^+^ T cells (1 × 10^6^ in 1 ml) were stimulated with anti-CD3 (1 μg/ml) and IL-2 (50 u/ml) in the absence or presence of 100 nM 1,25(OH)_2_D3 for 7 days. (a, c–d) *SERPINA1* and (b, c–d) *IL10* mRNA quantification by qPCR in 1,25(OH)2D3-treated cells was normalised to 18 s expression and is shown as a relative quantity (RQ) compared to expression of cells stimulated in the absence of 1,25(OH)_2_D3. (c–d) Each dot shows relative expression of both genes in individual (c) healthy and (d) PiZZ subjects. (a–b) * p < 0.05 as assessed by paired *t*-test. (c–d) Correlation between vitamin D regulation of SERPINA1 and IL-10 was assessed using a Pearson test (Healthy n = 9, PiZZ n = 10).

This work has identified that 1,25(OH)_2_D3-treatment promotes α-1-antitrypsin expression by CD4^+^ T cells, and furthermore that α-1-antitrypsin, at circulating levels, acts together with its binding partner, C3a, to promote IL-10 expression. We therefore next probed whether the α-1-antitrypsin-C3a axis may play an endogenous role in 1,25(OH)_2_D3-driven IL-10 expression by CD4^+^ T cells. Despite being a well-established effect of 1,25(OH)_2_D3, with evidence for in vivo relevance, this remains poorly understood and the kinetics indicate a likely indirect mechanism. To do so, we compared effects of 1,25(OH)_2_D3 on *SERPINA1* and *IL10* expression in CD4^+^ T cells from healthy volunteer donors with those from age-matched patients exhibiting a genetic mutation in the *SERPINA1* gene (PiZZ genotype), which can transcribe the *SERPINA1* gene, but cannot secrete the mutant, polymerised protein. As expected, *SERPINA1* mRNA levels were similarly induced by 1,25(OH)_2_D3 treatment in healthy control and PiZZ genotype CD4^+^ T cells (Fig. 5a). However, consistent with their genotype, reduced levels of α-1-antitrypsin protein synthesis by PiZZ CD4^+^ T cells compared to control individuals are routinely detected in our laboratory (Supplementary Fig. 3). Nevertheless, when the capacity of 1,25(OH)_2_D3 to enhance gene expression of *IL10* by CD4^+^ T cell cultures was compared in these two cohorts, PiZZ CD4^+^ T cells were found to have significantly lower abundance of *IL10* mRNA, which was indeed not upregulated compared to untreated cells (Fig. 5b). Additional analyses of the relationship between *SERPINA1* and *IL10* gene expression in 1,25(OH)_2_D3-treated CD4^+^ T cells identified a significant correlation was observed in cultures from healthy donors (r = 0.75; p = 0.02; n = 9), which was not observed in the α-1-antitrypsin-deficient patient cohort (r = 0.50; p = 0.14; n = 10) (Fig. 5c–d).

Taken together these data indicate that 1,25(OH)_2_D3 increases α-1-antitrypsin expression by CD4^+^ T cells, which then acts in turn to promote anti-inflammatory IL-10 expression by these cells. Additionally, we identify a novel interaction between α-1-antitrypsin and complement C3a, which is critical for IL-10 induction. The model is summarised in graphical form in Supplementary Fig. 4.

## 4 Discussion

This study identifies a novel interaction in the human immune system, between two ubiquitous mediators, α-1-antitrypsin and C3a, which directly bind to each other, and at physiological concentrations interact to drive anti-inflammatory IL-10 expression by CD4^+^ T cells. Additionally, we show that 1,25(OH)_2_D3 is a key upstream regulator in this axis, stimulating α-1-antitrypsin production by human CD4^+^ T cells – a novel cellular source of this protein.

A number of similarities exist in the anti-inflammatory function of α-1-antitrypsin and vitamin D. Deficiency of both α-1-antitrypsin and vitamin D are associated with inflammation and autoimmunity [20,21]. Additionally, the airway is an environment where the immune-modulatory effects of both α-1-antitrypsin and 1,25(OH)_2_D3 appear to be of key importance. Beyond its critical anti-elastase function, clearly evidenced by the development of COPD in deficient individuals, α-1-antitrypsin deficiency is also implicated in control of airway inflammation since asthma symptoms are also reported in α-1-antitrypsin deficiency [[22], [23], [24]]. Vitamin D deficiency is also implicated in airway immune homeostasis, as in respiratory diseases, including asthma, epidemiologic associations exist between vitamin D insufficiency and worse asthma control. Notably, the phenotype of vitamin D deficiency in severe asthma, and in particular treatment refractory asthma is associated with a neutrophilic profile, an enhanced pro-inflammatory Th17 phenotype and impaired induction of anti-inflammatory IL-10 [25]. Neutrophils are also proposed to play a prominent role in lung disease associated with α-1-antitrypsin deficiency, and our unpublished data demonstrate a significantly increased frequency of pro-inflammatory Th17 in these individuals (manuscript in preparation). As we already discuss in the introduction to this paper, DeMeo et al. [12] report that COPD progression in α-1-antitrypsin deficiency associated with IL-10 polymorphisms and α-1 antitrypsin replacement therapy increases circulating IL-10 in these individuals. Hence a number of clear parallels exist. The present study indicates that the similarity between α-1-antitrypsin and vitamin D deficiency may be explained by a biological link between these two mediators – specifically that 1,25(OH)_2_D3 regulates α-1-antitrypsin abundance and thereby its immune-modulatory properties (see model proposed in Supplementary Fig. 4). Further studies are now warranted to interrogate the importance of this axis in tissue environments and in the context of inflammation.

Previous reports have demonstrated anti-inflammatory actions of α-1-antitrypsin in vitro and in experimental models in vivo [9], with early papers for example reporting that α-1-antitrypsin therapy induces antigen-specific immune tolerance during islet allograft transplantation in mice [11,26]. The authors implicated a role for α-1-antitrypsin in maintaining dendritic cells in an immature state and in the induction of regulatory T cells (Treg), as assessed by increased mRNA for foxp3, cytotoxic T lymphocyte antigen-4, TGF-beta, IL-10, and IL-1 receptor antagonist. These functions closely mirror those described for vitamin D (1,25(OH)_2_D3) on immune cells [4,6,27]. In the present study we focused on the capacity of α-1-antitrypsin to enhance IL-10 synthesis by T cells since a major interest of the lab has been the control of IL-10 synthesis by 1,25(OH)_2_D3 [4,5]. A limitation of the current study is that we did not comprehensively address whether Foxp3 expression was also enhanced in these experiments, in part due to our earlier work showing that 1,25(OH)_2_D3 increases the frequency of distinct IL-10 or Foxp3 expressing CD4^+^ T cells, with little or no co-expression [3]. However, we did observe a reduced expression of *FOXP3* mRNA in CD4^+^ T cells obtained from PiZZ compared to control donors (data not shown: n = 5 per group; p = 0.0079). Furthermore, Mueller et al. [28] demonstrated that a single intramuscular administration of recombinant adeno-associated virus serotype 1 alpha-1 antitrypsin vector into alpha-1 deficient patients resulted in an active Treg response (approx. 10% of CD3^+^ cells were Foxp3 up to 5-years post administration, which was not seen in normal control muscle), suggesting future directions for our work.

The present study was able to compare responsiveness to 1,25(OH)_2_D3 in healthy control subjects versus those with a hereditary deficiency of α-1-antitrypsin protein, characterised by the PiZZ genotype, who possess around only 15% of normal wildtype circulating α-1-antitrypsin protein. Whilst CD4^+^ T cells from healthy subjects demonstrated a significant positive correlation between the capacity of 1,25(OH)_2_D3 to increase gene expression for IL10 and SERPINA1, this association was absent in the α-1-antitrypsin deficient donors. This comparison of the response to 1,25(OH)_2_D3 in α-1-antitrypsin deficient versus control subjects strengthen evidence implicating 1,25(OH)_2_D3 as an upstream regulator of this anti-inflammatory axis in these cells.

There appear to be a growing number of underlying mechanisms by which α-1-antitrypsin exerts immunomodulatory activity, which remain poorly understood (reviewed in 9). However, the anti-protease function of α-1-antitrypsin is not consistently reported to be required, and precedence exists for functions of α-1-antitrypsin that require binding to other serum components [9]. For example Bergin et al. [29] described the capacity of α-1-antitrypsin to regulate chemotaxis induced by soluble immune complexes and IL-8 by human neutrophils. Specifically, α-1-antitrypsin decreased the chemotactic response of CXCR1 signaling by binding IL-8 and inhibiting receptor engagement. In a very different example, additional protease-independent activity of α-1-antitrypsin was suggested by studies describing the induction by α-1-antitrypsin of angiopoietin-like protein 4 in human blood monocytes and lung microvascular endothelial cells [30]. This function of α-1-antitrypsin required binding to the fatty acids linoleic acid and oleic acid. Linoleic acid was also reported to enhance the capacity of α-1-antitrypsin to inhibit LPS-induced IL-1β synthesis by human neutrophils [31]. Thus our demonstration that induction of IL-10 in CD4^+^ T cells by α-1-antitrypsin is dependent on binding to the complement component C3a, adds to this growing body of evidence for protease-independent functions of α-1-antitrypsin.

C3a is shown to be critical to one of the major anti-inflammatory functions of α-1-antitrypsin. In addition to its key innate immune function, the complement system is now recognized to be involved in both the generation and regulation of adaptive immunity. C3a and C5a are essential for induction of inflammatory adaptive immune responses, signaling via their surface G-protein coupled receptors [17]. The complement system also promotes immune tolerance and drives IL-10, for example via ligation of CD46 [32]. The anti-inflammatory role we have identified for C3a in promoting IL-10 synthesis initially appears in contrast to its well-known pro-inflammatory effects, as highlighted by the impaired immunity associated with C3 deficiency [33]. However it is consistent with recent, apparently opposing observations of immune dysregulation,failure of immune tolerance mechanisms and allograft rejection in C3 and C3aR deficient animal models [34,35] and with observations in humans thatpurified CD4^+^ T cells from C3 deficient individuals demonstrate defective IL-10 responses in vitro [36]. Indeed, complement has been described as a “double-edged sword” in the regulation of immunity [37]. The dependency upon C3a for the anti-inflammatory effects of α-1-antitrypsin in vivo in disease models remains to be assessed using relevant transgenic systems.

Our study identifies 1,25(OH)_2_D3 as an upstream regulator of α-1-antitrypsin expression in human T cells. The major source of circulating α-1-antitrypsin is hepatocytes, although other cell types synthesise this protein. This includes monocyte-derived macrophages and dendritic cells, alveolar macrophages, bronchial epithelial cells and neutrophils, which may significantly contribute to local α-1-antitrypsin levels in the context of an immune response. However, to date we have found no evidence for regulation by 1,25(OH)_2_D3 of α-1-antitrypsin gene and/or protein levels in monocytes, respiratory epithelial cells or primary hepatocytes (data not shown). Our interpretation of these data is that this function of 1,25(OH)_2_D3 may therefore reflect an immunoregulatory–specific activity of 1,25(OH)_2_D3 in human T cells, adding to the growing list of such functions attributed to 1,25(OH)_2_D3 [1,38].

Growing recognition of vitamin D deficiency and insufficiency in recent years and its association with a wide range of immune-mediated pathologies, has meant that pharmacological vitamin D supplementation (with precursors of 1,25(OH)_2_D3) is now increasingly employed [1,38]. It is known to be safe, well-tolerated, relatively inexpensive and with a half-life of weeks. A number of clinical studies of vitamin D supplementation, with highly differing design and primary outcomes, have been performed. Notably, systematic review of these trials to date, indicate that vitamin D is beneficial in asthma [27]. Understanding the mechanisms whereby vitamin D acts to promote respiratory health are therefore likely to be important in improving clinical trial design. To date, the capacity of vitamin D to promote antimicrobial pathways, a common cause of asthma exacerbations, together with induction and/or maintenance of immune tolerance pathways are proposed [25]. In addition we, and others, have proposed that vitamin D beneficially modulates immune and clinical response to glucocorticoids. In early studies we demonstrated that 1,25(OH)_2_D3 restored the impaired steroid-induced IL-10 response in steroid refractory asthma patients both in vitro and in vivo [5], and clinical studies now provide evidence for modest steroid-sparing effects on vitamin D in asthma patients (reviewed in [25]). A very recent report of α-1-antitrypsin infusion therapy for steroid-resistant graft-versus-host disease suggested that α-1-antitrypsin was safe and potentially effective in treating these patients, and was associated with an increased ratio of regulatory to effector T cells following treatment [39], further demonstrating parallels with known effects of vitamin D.

α-1-antitrypsin has been used successfully in animal models of various autoimmune diseases as well as transplantation [9], and this has contributed to the growing interest in the therapeutic application of α-1-antitrypsin as an anti-inflammatory mediator, beyond its application in AAT deficiency. In addition to the above example in graft-versus-host disease, clinical studies of the safety and efficacy of α-1-antitrypsin treatment have been reported in conditions as diverse as type 1 diabetes [40], lung transplantation and acute myocardial infarction ([41]; see also clincialtrials.gov).

In summary, we have identified a novel, vitamin D (1,25(OH)_2_D3) controlled regulatory axis in the immune system, mediated through the interaction of α-1-antitrypsin and the complement anaphylatoxin C3a to stimulate IL-10 synthesis by CD4^+^ T cells. Our data highlight a novel potential therapeutic application for vitamin D in modulation of this axis, and suggest new roles for α-1-antitrypsin in the control of inflammatory disease. There is wide experience of the use of both vitamin D and α-1-antitrypsin as therapeutic agents in the US and Europe and both have very good safety profiles. Future trials offer the capacity to directly examine evidence for the role of α-1-antitrypsin in vitamin D-mediated immune regulation.

## Author contributions

Experimental work was largely carried out by SD and LR. HC, CC, PEP, MPN, DFR, and JR also contributed to experiments. PL supported analyses of transcriptomic datasets providing pilot observations upon which the experiments were conceived. JM and LR designed and performed all binding experiments. CK provided expertise on complement biology and identified that C3a alone regulates IL-10 in human CD4^+^ T cells [42]. The manuscript was prepared by SD, LR, BG and CMH, and all studies were conceived by SD, LR, BG, CK and CMH.
