# Supplementary material for: Vitamin D (1,25(OH)2D3) induces α-1-antitrypsin synthesis by CD4+ T cells, which is required for 1,25(OH)2D3-driven IL-10
Source: J Steroid Biochem Mol Biol. 2019 May;189:1–9. doi: 10.1016/j.jsbmb.2019.01.014 (PMC6525112; doi:10.1016/j.jsbmb.2019.01.014)
Supplement: Supplementary file 1 [file mmc1.docx]

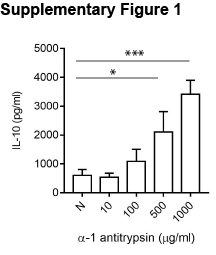


**Supplementary Figure 1: Human plasma-derived α-1-antitrypsin promotes production of IL-10 by human CD4^+^ T cells in a dose-dependent manner**

CD4^+^ T cells (1x10^6^ in 1ml) were stimulated with anti-CD3 (1μg/ml) and IL-2 (50u/ml) for 48 hours without or with the indicated concentration of human plasma-derived α-1-antitrypsin.

IL-10 levels were assessed by CBA and are summarised as mean ± SEM. p values derive from one-way ANOVA and Bonferroni’s paired post-test for multiple comparisons. * p < 0.05 and ** p < 0.01.


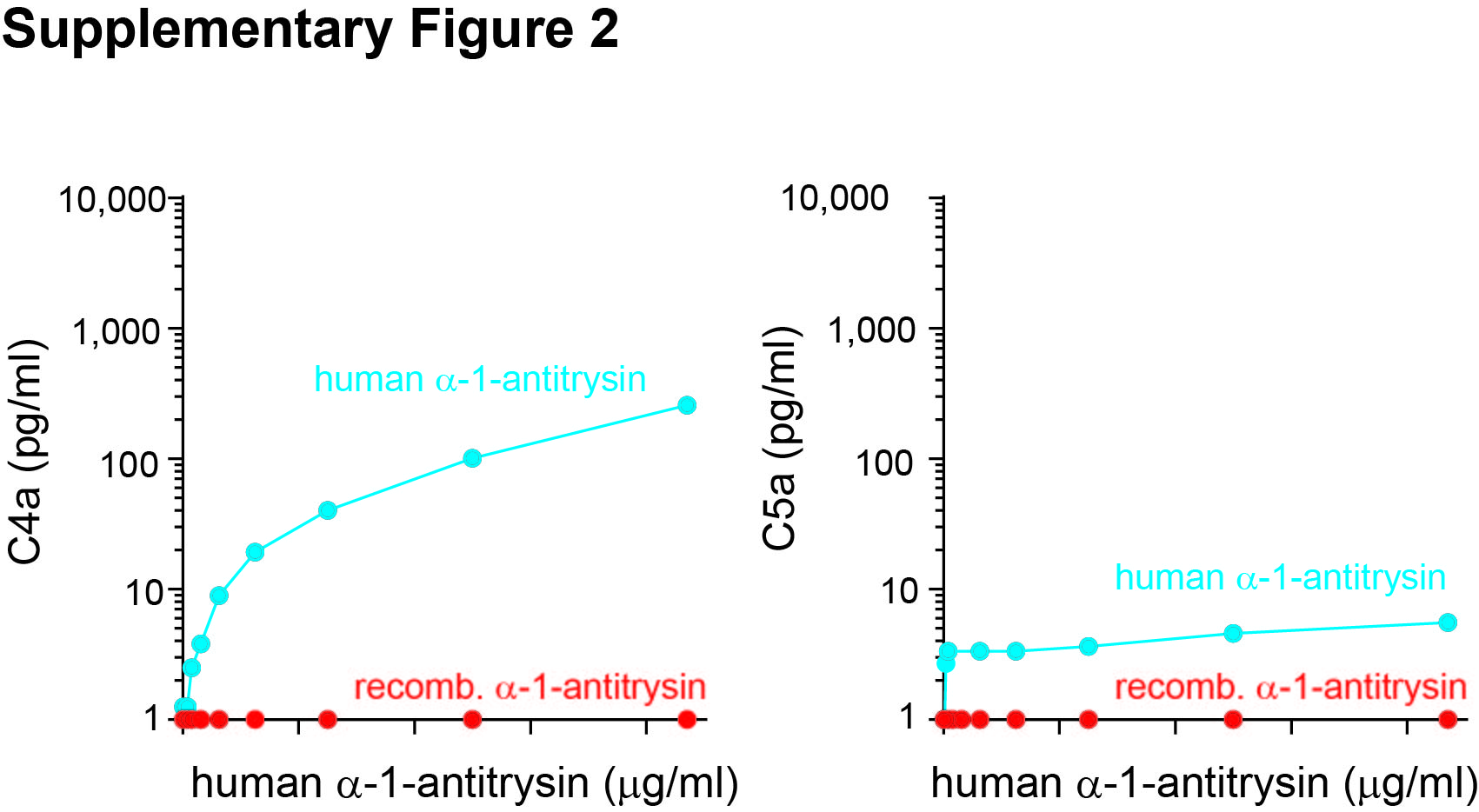


**Supplementary Figure 2: C4a and C5a content of α-1-antitrypsin preparations**

C4a and C5a content in human plasma-derived and recombinant α-1-antitrypsin was assessed by CBA anaphylatoxin flex set.

**
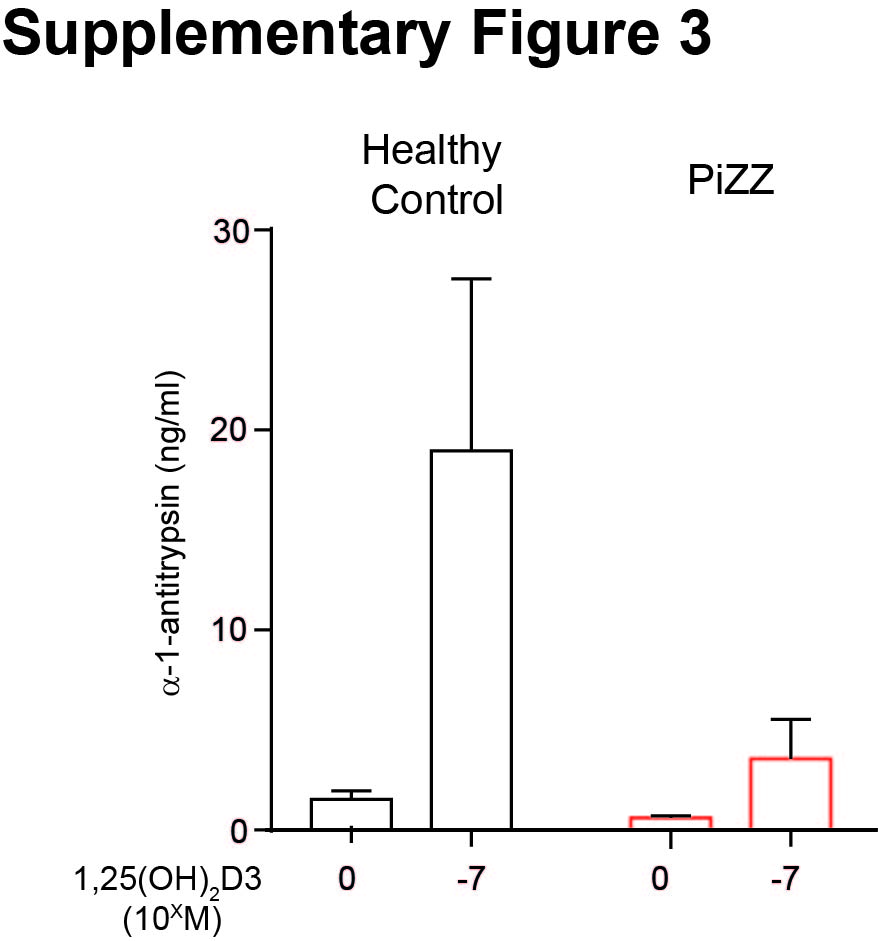
**

**Supplementary Figure 3: α-1-antitrypsin protein secretion by Healthy Control and PiZZ CD4+ T cells**

CD4^+^ T cells from healthy controls (n=17) or PiZZ individuals (n=20) (1x10^6^ in 1ml), in RPMI 10%FCS, were stimulated with anti-CD3 (1μg/ml) and IL-2 (50u/ml) for 48 hours in presence of 1,25(OH)_2_D3 as indicated. α-1-antitrypsin protein secretion was assessed by ELISA.

**
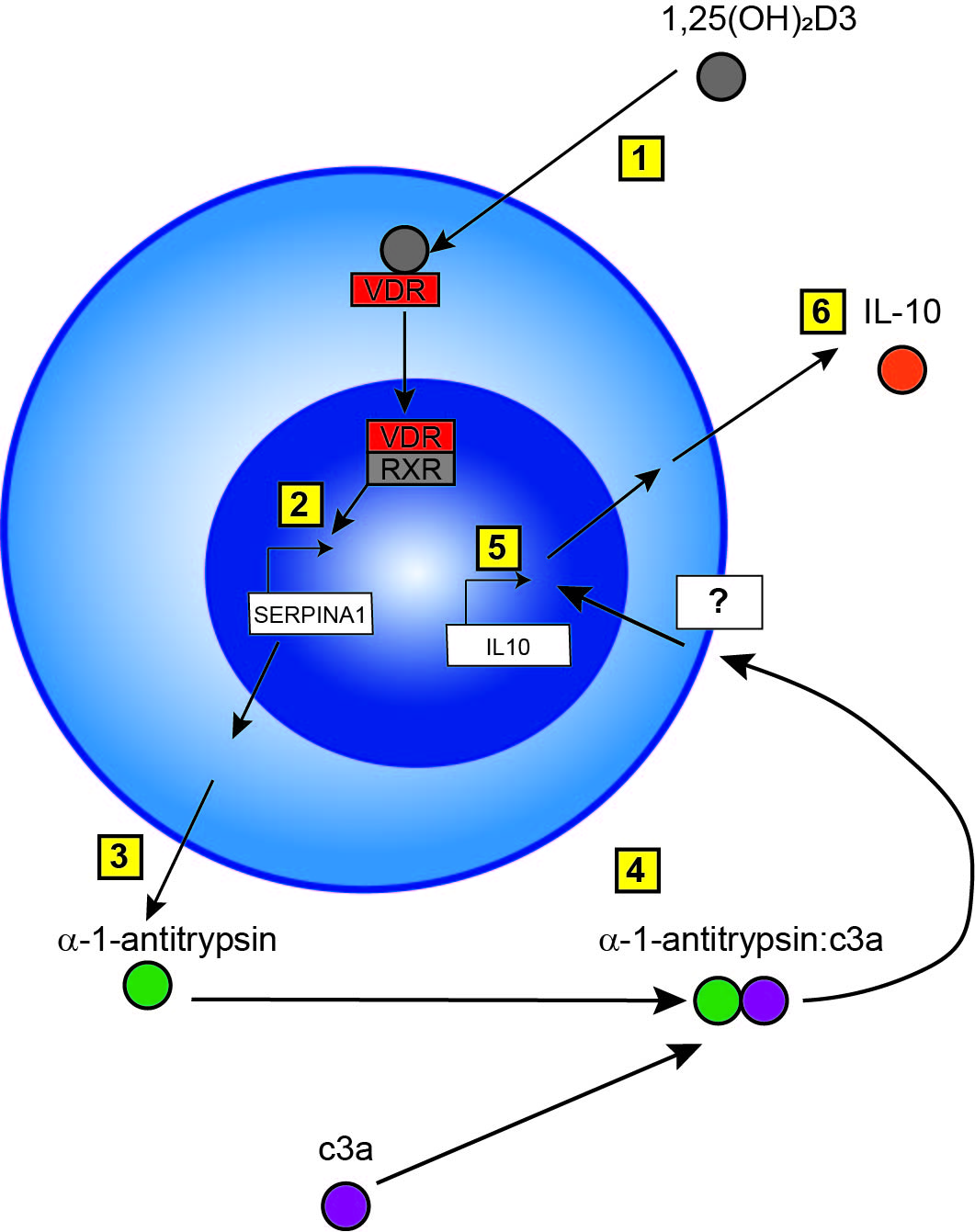
**

**Supplementary Figure 4: Graphical Representation**

The proposed model is as follows:

1) 1,25(OH)_2_D3 enters a CD4^+^ T cell and binds to its receptor, VDR. 2) VDR translocates to the nucleus where it interacts with its co-receptor RXR to drive transcription of SERPINA1 mRNA. 3) α-1-antitrypsin protein is translated and secreted by the CD4^+^ T cell. 4) α-1-antitrypsin binds with local c3a which may have been generated by the CD4^+^ T cell, other local immune cells or derive from the circulation. 5) The α-1-antitrypsin:c3a complex acts via an as-yet unknown pathway to drive transcription of IL-10 mRNA. 6) IL-10 protein is translated and secreted by the CD4^+^ T cell
